# Prognostic marker identification based on weighted gene co-expression network analysis and associated *in vitro* confirmation in gastric cancer

**DOI:** 10.1080/21655979.2021.1957645

**Published:** 2021-08-02

**Authors:** Haonan Guo, Jun Yang, Shanshan Liu, Tao Qin, Qianwen Zhao, Xianliang Hou, Lei Ren

**Affiliations:** a Department of Clinical Laboratory, The Affiliated Hospital of Guilin Medical University, Guangxi, Guilin, China; b Central Laboratory, Guangxi Health Commission Key Laboratory of Glucose and Lipid Metabolism Disorders, The Second Affiliated Hospital of Guilin Medical University, Guilin, Guangxi, China

**Keywords:** Gastric cancer, WGCNA, *CEMIP*, prognostic biomarker, tumor infiltration

## Abstract

The aim of this study was to explore the potential molecular mechanisms of Gastric cancer (GC) and identify new prognostic markers for GC. RNA sequencing data were downloaded from the Gene Expression Omnibus database, and 418 differentially expressed genes (DEGs) were screened. Weighted correlation network analysis (WGCNA) was performed to identify six hub modules related to the clinical features of GC. Cytoscape software was used to identify five hub genes in the co-expression network, including CST1, CEMIP, COL8A1, PMEPA1, and MSLN. The TCGA database was used to verify hub gene expression in GC. The overall survival in the high CEMIP expression group was significantly lower than that of patients in the low CEMIP expression group. CEMIP expression was also found to be negatively correlated with B cell and CD4 + T cell infiltration. Further, associated in vitro experiments confirmed that CEMIP downregulation suppressed the proliferation and migration of GC cells and impaired the chemoresistance of GC cells to 5-fluorouracil.

Our study effectively identified and validated prognostic biomarkers for GC, laying a new foundation for the therapeutic target, occurrence, and development of gastric cancer.

## Introduction

Gastric cancer (GC) is one of the most common malignancies of the digestive system. According to the latest statistics of the World Health Organization (WHO), the incidence and mortality rates of GC in 2018 ranked fifth and third, respectively, among all malignant tumors [1]. In 2020, the United States expected 27,600 new cases and 11,010 deaths from GC [2]. The high incidence and mortality rates in GC has made it a public health concern, especially in developing countries [3]. Although recent advances in endoscopic techniques, imaging techniques, surgical techniques, and the use of targeted drugs have extended the overall survival of patients with GC to a certain extent, most patients already have advanced GC by the time they are diagnosed, and the 5-year overall survival rate for patients with the disease is only 5% [4]. Therefore, it is important to find relevant biomarkers that have good predictive accuracy for use in the early detection of GC, to screen appropriate patients who can receive targeted therapies for GC, and for the accurate prediction of GC prognoses.

The rapid development and widespread use of high-throughput technologies has produced a large amount of gene expression profile data and made them publicly available, where many studies have identified DEGs by comparing gene expression data from tumor and para-cancerous tissues [5]. However, previous research has focused more on the role of single DEGs than on the complex connections between DEGs. Weighted correlation network analysis (WGCNA) based on RNA sequencing data can be used to mine functional gene modules and identify hub genes that could be potential cancer biomarkers and therapeutic targets [6]. Previous studies have mined public databases for potential prognostic biomarkers in GC. For example, Zhou et al. have identified 14 independent prognostic DEGs in gastric cancer and have constructed a prognostic signature with good predictive performance [7]. Yang et al. used data from public databases to develop a prognostic prediction model of GC based on cGAS-STING pathway-related genes (CSRs) [8].

However, these studies have not explored the potential mechanisms of the target genes they identified in the development of GC, and no relevant experiments were performed to verify their conclusions based on their bioinformatics analyses. The advantage of this study lies in the combination of weighted gene co-expression network analysis to identify the biologically significant genes that are related to clinical characteristics, and validation experiments were conducted to improve the accuracy and clinical applicability of the results obtained.

The purpose of this study is to identify the core genes associated with the pathogenesis of GC by integrating multiple GEO data sets and WGCNA algorithms. Finally, we focused on one core gene, *CEMIP*. We observed that the expression of *CEMIP* was negatively correlated with the infiltration of B cells and CD4 + T tumor cells and that it is an independent risk factor for the prognosis of patients with GC. Further, the validation experiments we performed confirmed that *CEMIP* downregulation suppressed the proliferation and migration of GC cells and impaired the chemoresistance of GC cells to 5-fluorouracil (5-FU). Therefore, *CEMIP* may be a new prognostic biomarker and therapeutic target for GC.

## Materials and methods

### Flow chart

**Figure 1.**
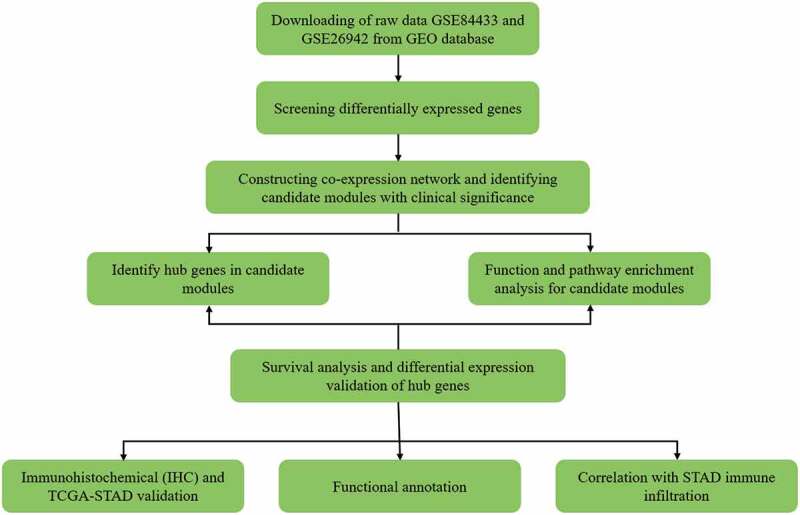
Workflow diagram

Figure 1 shows the workflow of this study.

## Data sourcing and preprocessing

The GSE84433 and GSE26942 data sets in the GEO database were selected for subsequent analyses [9]. The data type of each group was set as ‘expression profiling by array,’ and the species was ‘*Homo sapiens*.’ The GSE84433 data set annotation platform was GPL6947, and it included 357 GC samples. The GSE26942 data set annotation platform was GPL570, and it included 12 normal tissue samples and 205 GC samples. The two data sets underwent batch correction procedures [10]. Data on GC and normal tissue samples obtained from the TCGA database were downloaded to evaluate the influence of the hub genes on GC prognosis.

## Identification of DEGs

Using the limma package [11] in R v4.0.0, the DEGs between the control and GC groups were identified. The threshold value was set to |log(fold change)| >2, p < 0.01 to identify the DEGs. According to the mRNA expression levels of the different samples, they were analyzed via hierarchical cluster analysis using the pheatmap package in R.

## Construction of the WGCNA network

To select the DEGs and construct the corresponding WGCNA network, the outliers were removed and the remaining samples were used to construct a co-expression network under the premise of ensuring the reliability of the network structure. The pickSoftThreshold function of the WGCNA was used to identify the appropriate soft threshold (β) value to obtain a scale-free network. A hierarchical clustering tree was constructed based on the cluster analysis results. Gene modules were obtained via dynamic tree cutting. The depth segmentation parameter was set to 2, and the minimum size cutoff was set to 10. Highly similar modules were clustered together with a height cutoff value of 0.25.

## Identification of modules with clinical significance

Two parameters, including module characteristic gene (ME) and gene significance (GS), were used to distinguish the modules related to the clinical features of GC. The ME parameter was a major component of each module. Correlations between the ME and clinical features were determined using Pearson chi-squared tests, where we screened for highly relevant modules (p < 0.05). The GS parameter represented the expression of each gene and its degree of correlation with a clinical feature. The module member (MM) measured the correlation between each gene in the module and the ME of the module. By analyzing the relevance between the GS and MM, modules with significant clinical significance were further screened out.

## Function and pathway enrichment analyses

A Gene Ontology (GO) function analysis and Kyoto Encyclopedia of Genes and Genomes (KEGG) pathway enrichment analysis were performed to explore the potential molecular mechanisms these modules are involved in (p < 0.05).

## Identification and verification of the hub genes in the key modules

Hub genes with high connectivity were identified using the cytoHubba plug-in of Cytoscape v3.7.0 [12]. The genes in the network were arranged from high degree scores to low degree scores. The five nodes with the highest scores were chosen as the candidate hub genes.

We also conducted survival analysis using Kaplan-Meier Plotter (http://kmplot.com) to determine whether each hub gene was associated with prognosis [13]. GEPIA (http://gepia.cancerpku.cn/) is an online database designed to analyze gene expression in normal and cancerous tissues [14]. In addition, the differentially expressed hub genes between the different histological types of GC tissues and normal tissues were analyzed using the Oncomine database (https://www.oncomine.org/resource/login.html#) [15]. Differences in gene expression levels were considered statistically significant when the p-value was <0.05. The differentially expressed hub genes between GC tissues and normal tissues were analyzed using the Human Protein Atlas (HPA).

## LinkedOmics database

The LinkedOmics database [16] contains multi-group and clinical data of 11,158 patients with 32 cancer types. The co-expressed genes were statistically analyzed using the Pearson correlation coefficient, and volcano and heat maps were constructed.

## TIMER database analysis

The TIMER database is an effective tool to infer the abundance of tumor-infiltrating immune cells from the expression profiles of target genes [17]. The TIMER database contains 897 samples of 32 cancer types from the TCGA database, thus the clinical characteristics of different immune cells in different types of cancer can be analyzed. The infiltration levels of six types of immune cells (B cells, macrophages, CD8 + T cells, CD4 + T cells, dendritic cells, and neutrophils) can be calculated using this tool. Using the ‘gene’ module, correlations between hub gene expression and immune cell infiltration were analyzed using Spearman’s correlation. Furthermore, the ‘survival’ module was used to construct Kaplan-Meier diagrams related to the hub genes and show the relationship between immune cell infiltration and hub gene expression.

## Flow cytometry

Cells were washed with phosphate-buffered saline (PBS) after being digested with trypsin, then an Annexin V-FITC Apoptosis Detection Kit (Beyotime) was used to detect cell apoptosis, according to the manufacturer’s instructions. Apoptotic cells were dual-stained with annexin V-fluorescein isothiocyanate (FITC) and propidium iodide (PI) using an Annexin V/FITC Kit (Thermo Scientific, Shanghai, China). A BDTM LSR II flow cytometer (BD Biosciences, San Jose, CA, USA) was used to sort the cells according to their apoptosis status and CellQuest software (BD Biosciences) was used for data analysis.

## Wound healing and Transwell assays

For the wound-healing assay, cells were seeded on six-well plates and cultured until 95% confluence. A sterile 200-μL plastic pipette tip was used to scratch the cell monolayer gently. The wound was then photographed. The wound was photographed again after 24 h. For the Transwell assays, 2 × 10^4^ cells suspended in serum-free medium were seeded in the upper chamber membrane, which was or was not coated with Matrigel (BD Biosciences) for the Transwell migration and invasion assays, respectively. The lower chamber was filled with 500 μL of a culture medium containing 10% fetal bovine serum. After 24 h of incubation, the underside of the membrane was fixed for 30 minutes and stained with 0.1% crystal violet. A cotton swab was used to wipe the inner side of the membrane. The number of cells that migrated were then quantified under a microscope.

## Western blot analysis

Cancer cells were collected and lysed in NP-40 lysis buffer for 30 min at 4°C after washing twice with cold PBS. A bicinchoninic acid assay kit (Thermo) was used to measure the total protein concentration. Protein extracts were separated via electrophoresis in pre-made 8–12% sodium dodecyl sulfate-polyacrylamide gels containing tris(hydroxymethyl)aminomethane hydrochloride and then transferred to a polyvinylidene difluoride membrane. The membrane was further incubated with the indicated antibodies and detected using the chemiluminescence method.

## Results

From the analyses we performed, 418 DEGs were identified using the GSE84433 and GSE26942 data sets, of which 53 were upregulated and 365 were downregulated. WGCNA was then used to identify the mRNA modules related to the clinical features of GC and six gene modules were obtained. The blue module was positively correlated with adjuvant chemotherapy, tumor type, tumor location, and overall survival, and the genes in this module were mainly involved in the biological processes of cell adhesion, multicellular organism development, cellular differentiation, among others. The degree of each node in the blue module was calculated using Cytoscape software. The five nodes with the highest scores were selected as the candidate genes: *CST1, CEMIP, COL8A1, PMEPA1*, and *MSLN*. The high expression level of these five hub genes was associated with poor prognosis in GC. The prognostic value of *CEMIP* was further validated using the TCGA database. The overall survival of patients in the high *CEMIP* expression group was significantly lower than those in the low *CEMIP* expression group. *CEMIP* expression was also negatively correlated with B cell and CD4 + T cell infiltration. Further, our experiments confirmed that *CEMIP* downregulation suppressed the proliferation and migration of GC cells and impaired the chemoresistance of GC cells to 5-fluorouracil. Hence, *CEMIP* may become a new prognostic biomarker and therapeutic target for GC.

### Database analyses showed that 418 DEGs were identified between the normal group and the GC group

**Figure 2.**
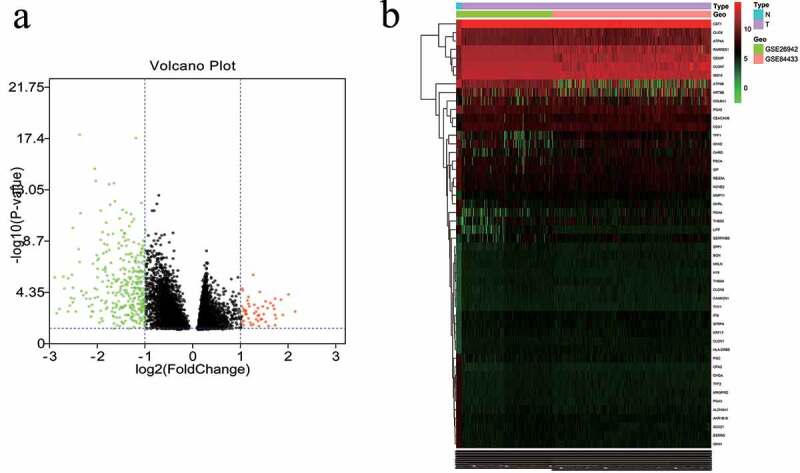
Differentially expressed mRNAs in the first 50 genes identified in the GEO database. (a) Volcano map of the mRNA expression values between gastric cancer samples and adjacent normal samples (red represents upregulated genes, green represents downregulated genes). (b) Hierarchical clustering heat map of differentially expressed mRNAs

The GSE84433 and GSE26942 data sets included 12 normal controls, 574 GC samples, and 25,077 genes. Differences in the mRNA expression levels between the GC and control groups were then obtained. It was found that there were 418 DEGs in the GC samples when compared with the control samples, 53 of which were upregulated and 365 were downregulated. Heatmaps and volcano plots were constructed to present these DEGs (Figure 2).

### Five hub genes were identified based on WGCNA

**Figure 3.**
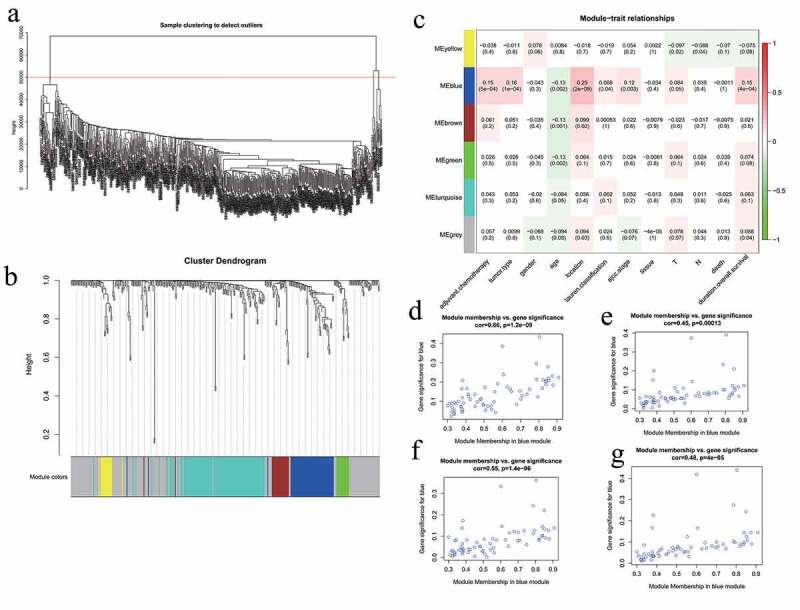
WGCNA analysis. (a) Sample hierarchical clustering map. (b) Gene tree (top) and gene modules with different colors (bottom). (c) Heat map showing correlations between the modules and clinical features of GC. The numbers represent the correlations, with red showing positive correlations and green showing negative correlations. (d) Scatter plot of the correlations between the blue module genes and the tumor location. (e) Scatter plot of the correlations between the blue module genes and adjuvant chemotherapy. (f) Scatter plot of the correlations between the blue module genes and overall survival. (g) Scatter plot of the correlations between the blue module genes and tumor type

**Figure 4.**
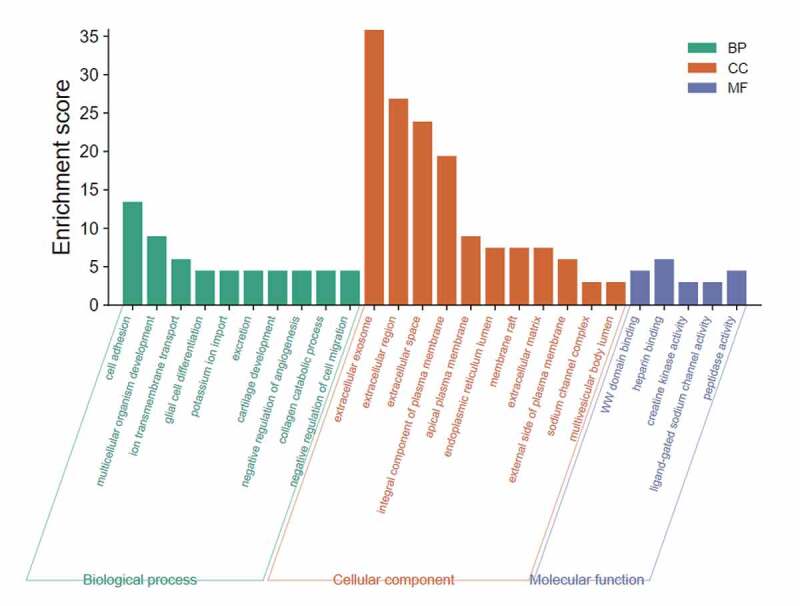
Enrichment analysis of the blue module genes. The length of the bar represents the enrichment score. A higher score means that more genes are enriched

**Figure 5.**
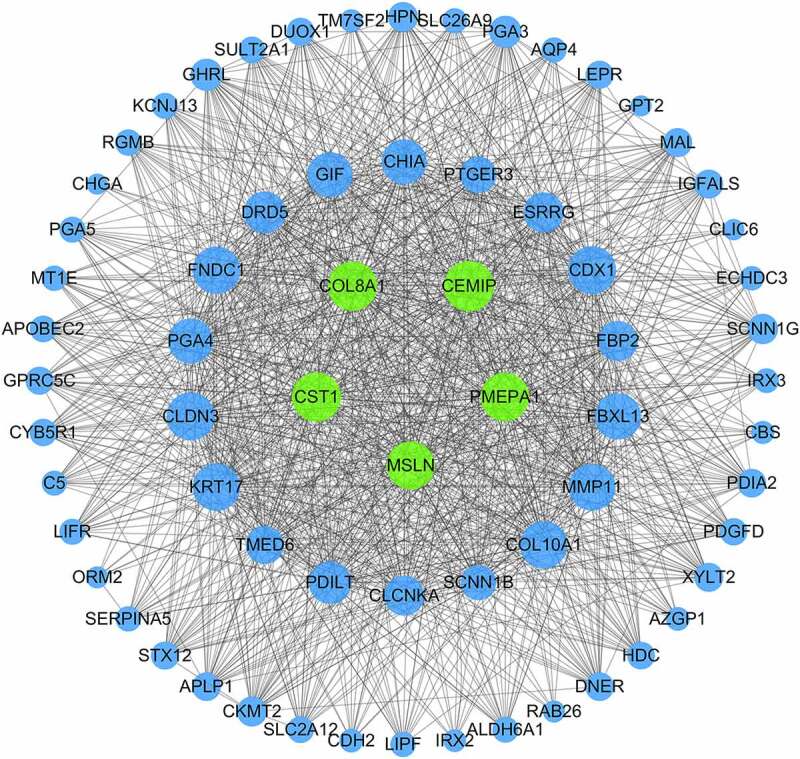
Gene network of the blue module genes. The blue nodes represent the genes in the blue module and the gray lines represent the co-expression relationships between these genes. The larger the blue node, the higher its degree score. Green represents the hub genes, and blue represents the non-hub genes

The flashClust package in R was used to perform a hierarchical clustering of the selected genes, as shown in Figure 3(a). A total of 11 outlier samples were removed, and the remaining 558 samples were used to construct a co-expression network. β-values from 1 to 20 were used to calculate the independence and average connectivity between samples. When β = 4, the independence was greater than 0.85, and the corresponding average connectivity was close to 0, indicating that the network satisfied the requirements of scale-free network distribution. The adjacent gene modules were identified and combined using the dynamic tree cutting method. Finally, we obtained six gene modules. The gray module included genes that were not co-expressed with other genes (Figure 3(b)). By correlating the gene modules with the clinical features, we selected the module that was significantly associated with certain clinical features (Figure 3(c)). The blue module was positively correlated with adjuvant chemotherapy, tumor type, tumor location, and overall survival (p = 5e-04, p = 1e-04, p = 2e-09, and p = 4e-04, respectively). Further analysis showed that there was a significant correlation between the GS and MM in the blue module (cor = 0.45, p = 0.00013; cor = 0.48, p = 4e-05; cor = 0.66, p = 1.2e-09; cor = 0.55, p = 1.4e-06). Thus, the blue module may be associated with GC (Figure 3(d-g)). GO enrichment analysis was performed to determine the biological significance of the genes in the blue module. The results showed that the genes in this module mainly involved the biological processes of cell adhesion, multicellular organism development, cellular differentiation, among others (Figure 4). The degree of each node in the blue module was calculated using Cytoscape software. The five nodes with the highest scores were selected as the candidate genes: *CST1, CEMIP, COL8A1, PMEPA1*, and *MSLN*. Figure 5 shows the blue module network.

## Expression and clinical significance of *CST1, CEMIP, COL8A1, PMEPA1*, and *MSLN* in GC

**Figure 6.**
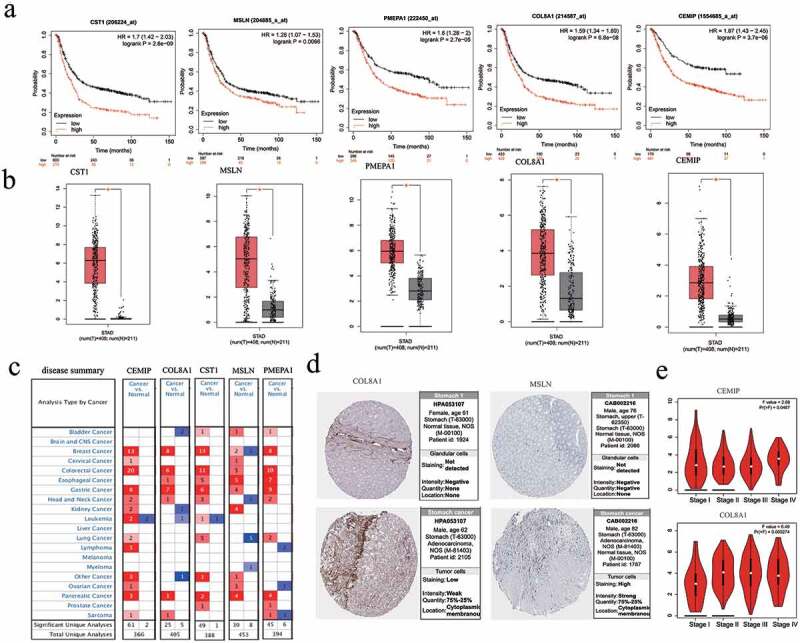
Survival analysis and differential expression of the hub genes. (a) Survival analysis of the hub genes in gastric cancer. (b) Analysis of the differential expression of the hub genes using the GEPIA database. The box diagram shows the mRNA expression values of the hub genes in gastric cancer tissues (red) and normal tissues (gray). *p < 0.05. (c) Analysis of the differential expression of the hub genes in different tumors using the Oncomine database. (d) Protein expression of the hub genes between normal and tumor tissues. (e) Expression of *CEMIP* and *COL8A1* in different stages of gastric cancer from the HPA database

Survival analysis showed that the high expression of the obtained core genes *CST1, CEMIP, COL8A1, PMEPA1*, and *MSLN* was associated with poor prognosis (p < 0.05; Figure 6(a)). The expression of these genes in GC tissues was significantly higher than in normal tissues (p < 0.05; Figure 6(b)). By setting the p-value to < 0.01, the |log(fold change)| to > 2, and the gene rank to 10%, the expression of each of these five hub genes in different types of cancer were analyzed. The results showed that *CST1, CEMIP, COL8A1, PMEPA1*, and *MSLN* were upregulated in most cancers and were downregulated in a few (Figure 6(c)). According to the HPA database, the five hub genes were upregulated in GC tissues (Figure 6(d)). The expression of *CEMIP* and *COL8A1* in different stages of GC was also found to be significantly different (Figure 6(e)).

### The prognosis prediction value of CEMIP was verified using the TCGA database

**Figure 7.**
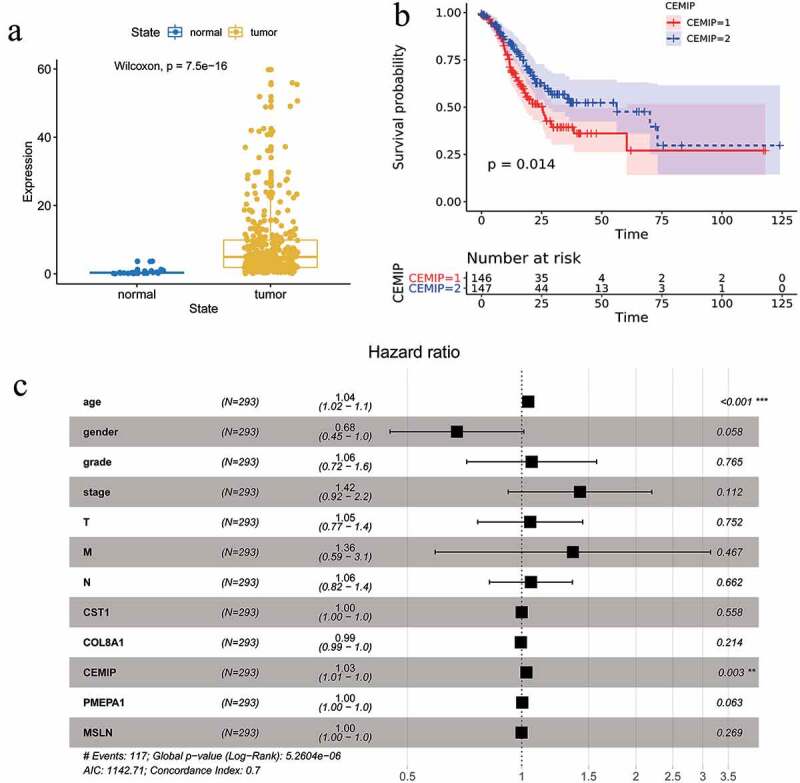
(a) Validation of the differential expression of the hub genes in gastric cancer and para-cancerous normal samples based on the TCGA database. (b) Validation of the effect of the hub genes on prognosis based on the TCGA database. (c) Multivariate analysis showing that age and *CEMIP* expression were independent risk factors for the prognosis of GC

At present, there are only a few studies regarding the expression and relationship of *CEMIP* in GC, so we analyzed this gene in depth. We downloaded the gene expression data and related clinical information of the TCGA-STAD cohort. We found that the expression of *CEMIP* in GC tissues was significantly higher than in normal tissues (Figure 7(a)). Taking the median *CEMIP* expression as the threshold, half of the samples with the higher *CEMIP* expression was considered as the high-expression group, and the other half was considered the low-expression group. Survival analysis showed that the overall survival of the high-expression group was significantly lower than that of the low-expression group (Figure 7(b)). Multivariate Cox analysis suggested that age (HR = 1.04, p < 0.05) and high *CEMIP* expression (HR = 1.03, p < 0.05) were independent risk factors for prognosis in patients with GC (Figure 7(c)).

### Molecular mechanisms of CEMIP in GC

**Figure 8.**
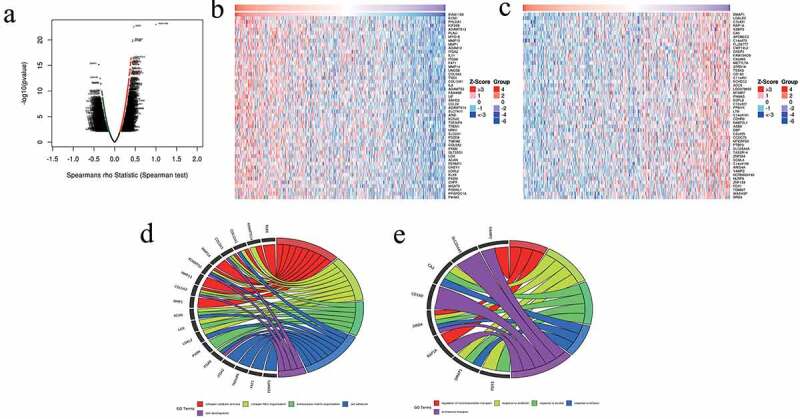
Co-expressed genes in STAD as determined from the LinkedOmics database. (a) *CEMIP* and the highly relevant genes identified from the Pearson’s chi-squared tests obtained from STAD samples. (b) Heat maps showing the top 50 genes in STAD that were positively associated with *CEMIP*. (c) Heat maps showing the top 50 genes in STAD that were negatively associated with *CEMIP*. (d) GO enrichment analysis results showing the top 50 genes that were positively correlated with *CEMIP*. (e) GO enrichment analysis results showing the top 50 genes that were negatively associated with *CEMIP.*

To investigate the potential biological roles and molecular mechanisms of *CEMIP* in GC, we analyzed the genes co-expressed with *CEMIP* in GC using LinkedOmics. A total of 8,562 genes (dark red dots in Figure 8(a)) were significantly positively correlated with *CEMIP*, while 11,664 genes (dark green dots in Figure 8(a)) were significantly negatively correlated with *CEMIP*. We then selected the top 50 genes that were positively and negatively associated with *CEMIP* (Figure 8(b and c), respectively). GO enrichment analysis showed that the genes positively correlated with *CEMIP* were mainly involved in the collagen catabolic process and collagen fibril organization (Figure 8(d)). The genes negatively associated with *CEMIP* were involved in the regulation of neurotransmitter transport, response to antibiotics, and other life processes (Figure 8(e)).

### Correlation between CEMIP expression and the immune microenvironment

**Figure 9.**
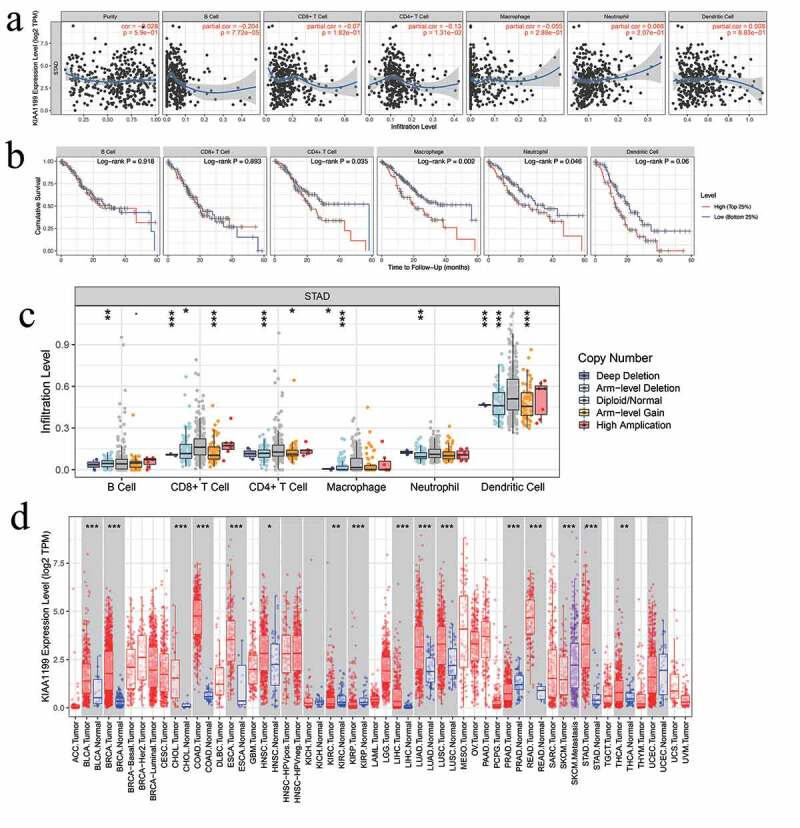
Correlations between *CEMIP* expression and different immune cells. (a) Relationships between *CEMIP* expression and the presence of six immune cells (B cells, CD4 + T cells, CD8 + T cells, macrophages, neutrophils, and dendritic cells) in STAD. (b) Kaplan-Meier curves related to the six immune cells in the STAD cohort. (c) *CEMIP* copy number analysis showing that it affects the levels of B cells, CD8 + T cells, macrophages, and dendritic cells in STAD. * p < 0.05, ** p < 0.01, and *** p < 0.001. (d) Expression of *CEMIP* across several cancers. * p < 0.05, ** p < 0.01, and *** p < 0.001

To explore the potential relationship between *CEMIP* and the immune microenvironment in GC, we evaluated the relationship between *CEMIP* expression and immune cell invasion in the TCGA-STAD cohort using the TIMER database. *CEMIP* expression was negatively correlated with B cell and CD4 + T cell infiltration in patients with stomach adenocarcinoma (STAD) (r = −0.2042, p = 7.72e-05 and r = −0.13, p = 1.31e-0, respectively; Figure 9(a)). In addition, high macrophage infiltration in patients with STAD was significantly associated with poor prognosis (p = 0.0042; Figure 9(b)). The copy number variation of *CEMIP* was also significantly correlated with the infiltration of CD4 + T cells, B cells, CD8 + T cells, neutrophils, macrophages, and dendritic cells (Figure 9(c)). In addition to being upregulated in STAD, *CEMIP* expression was also upregulated in a variety of cancers, including lung adenocarcinoma, colon adenocarcinoma, and head and neck squamous cell carcinoma (Figure 9(d)).

### Suppression of CEMIP expression inhibited the migration and invasion capacity of GC cells and chemoresistance to 5-FU

**Figure 10.**
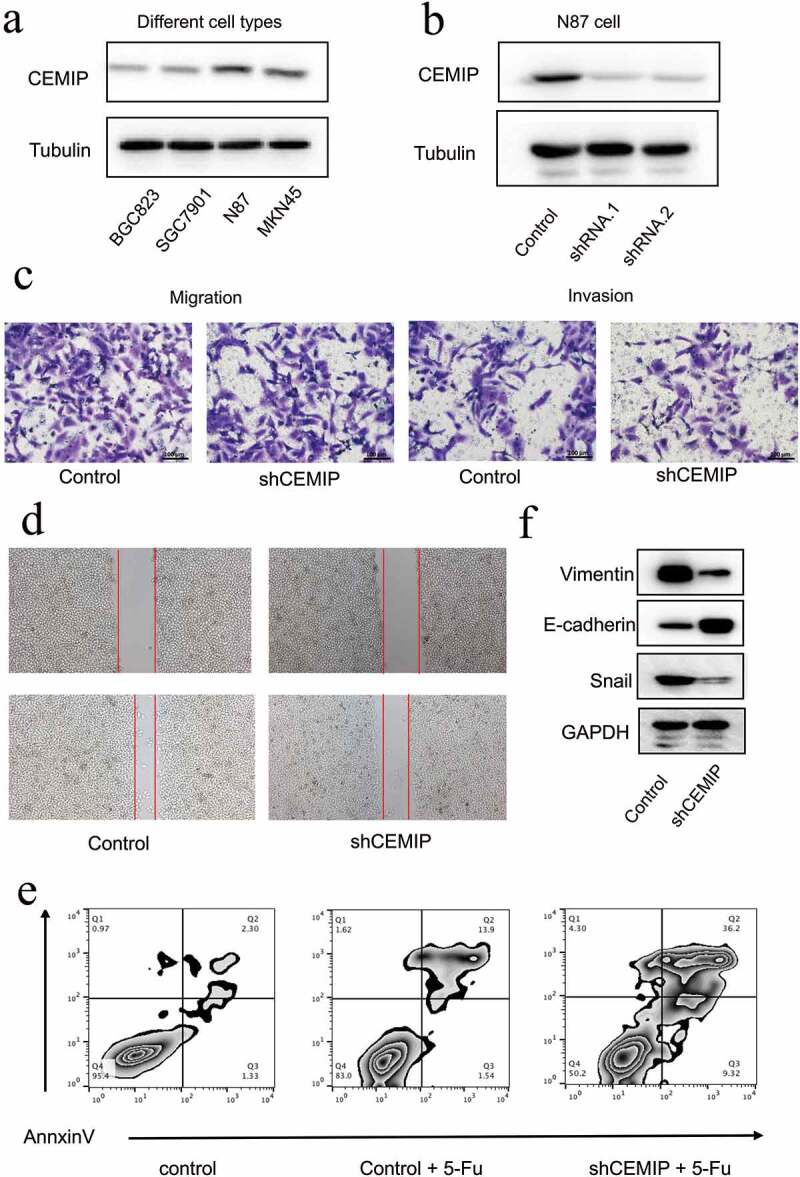
Biological behavior of *CEMIP* in gastric cancer *in vitro*. (a) Baseline expression of *CEMIP* in four kinds of cancer cells. (b) Two shRNAs that target *CEMIP* were used to knock down *CEMIP* expression, and its effect was confirmed via western blot analysis. (c, d) The effect of *CEMIP* on cell migration and invasion was studied using Transwell and wound healing assays, respectively. (e) The effect of *CEMIP* on chemoresistance to 5-FU. (f) EMT-associated markers, such as Vimentin and Snail, were upregulated after knocking down the expression of *CEMIP.*

The results of the bioinformatics analyses were further confirmed in *in vitro* experiments. The baseline expression of *CEMIP* was evaluated in four GC cell lines: BGC823, SGC7901, N87, and MKN45 (Figure 10(a)). Western blot analysis results showed that the baseline expression of CEMIP was the highest in the N87 cell line, which was used for subsequent experiments.

To investigate the cellular function of *CEMIP*, two shRNAs were used to knock down *CEMIP* expression, and their effect was confirmed via western blot analysis (Figure 10(b)). The results showed that CEMIP expression was knocked down successfully. To investigate the function of *CEMIP* in terms of cell migration and invasion, Transwell experiments were performed. After the knockdown of *CEMIP*, the migration and invasion capacity of GC cells was suppressed (Figure 10(c)). These results were further confirmed through a wound-healing assay (Figure 10(d)). After knocking down *CEMIP* expression, the migration of GC cells was impaired significantly. The underlying mechanism for this was confirmed via western blot analysis. Epithelial-mesenchymal transition (EMT) is an important phenomenon observed in cancer progression and is associated with the metastatic transformation of tumor cells. EMT-associated markers, such as Vimentin and Snail, were upregulated after *CEMIP* was knocked down (Figure 10(f)), suggesting that *CEMIP* may promote GC cell migration by promoting EMT in GC cells.

The molecule 5-FU is currently used as a chemotherapeutic agent in several cancers, including GC. Thus, we studied the role of *CEMIP* on the acquisition of chemoresistance to 5-FU. After the downregulation of *CEMIP*, the number of apoptosis of cancer cells increased, suggesting that the overexpression of *CEMIP* promoted the chemoresistance of GC cells to 5-FU (Figure 10(e)).

## Discussion

GC is a common malignancy that has caused considerable morbidity and mortality. In recent years, the use of PD-1 and PD-L1 checkpoint inhibitors have become the preferred immunotherapeutic strategy for melanoma [18], glioblastoma multiforme [19], and hepatocellular carcinoma [20]. However, due to the highly heterogeneous nature of GC, the efficacy of immunotherapy drugs and the responsiveness of patients to these novel kinds of treatments vary considerably [21]. Therefore, it is of great importance to reveal the potential molecular mechanisms governing the initiation and development of GC, as well as to discover novel prognostic markers and potential therapeutic targets to improve the survival outcomes of patients with GC.

In this study, WGCNA was used to comprehensively analyze the screened DEGs in GC. The blue module genes were significantly positively correlated with adjuvant chemotherapy, tumor type, tumor location, and overall survival. This module mainly involved key biological processes including cell adhesion, multicellular organism development, glial cell differentiation, among others.

The degree of each node in the blue module was calculated and the five nodes with the highest scores were selected as the candidate genes. Five hub genes (*CST1, CEMIP, COL8A1, PMEPA1*, and *MSLN*) were identified. Cystatin 1 (CST1) is a member of the cystatin superfamily and functions by inhibiting the proteolytic activity of cysteine protease [22]. *CST1* was found to be highly expressed in GC. The knockdown of *CST1* reduced the proliferation of GC cells and increased the proteolytic activity of cathepsin. Therefore, CST1 can be used as a tumor marker for GC [23–26]. The protein HOXC10 directly binds to the promoter region of *CST1* to promote the proliferation and migration of GC cells [27]. The gene *COL8A1* is highly expressed in GC and is associated with shorter overall survival. Collagen, type VIII, alpha 1 (COL8A1) participates in the malignant biological behavior of GC by promoting the proliferation of GC cells and inhibiting their apoptosis [28]. The transmembrane prostate androgen-inducible protein 1 (PMEPA1) is a single-pass transmembrane protein that is functionally involved in the TGF-β signaling pathway. *PMEPA1* expression in the malignant tissues of GC has been observed to be significantly upregulated [29]. Mesothelin (MSLN) can be targeted by chimeric antigen receptor (CAR) T cells, and anti-MSLN CAR T cells have been used in the treatment of GC [30].

Cell migration-inducing and hyaluronan-binding protein (CEMIP) is a Wnt-related protein involved in memory and synapse formation, as well as in cancer and inflammatory processes. There are only a few studies on the role of CEMIP in GC [31], although it has already been demonstrated that CEMIP may promote the metastasis of GC cells by activating related signaling pathways [32,33]. However, these previous studies usually have limitations due to their small sample sizes. Here, we used the TCGA-STAD cohort to confirm the high expression of *CEMIP* in GC. The mean overall survival of patients in the high-expression group was significantly lower than those in the low-expression group. Previously, CEMIP was also found to participate in the degradation of hyaluronic acid in rheumatoid arthritis [34]. In idiopathic pulmonary fibrosis, *CEMIP* silencing reduced the production of collagen, thereby reducing the ability of lung fibroblasts to proliferate and migrate [35]. In this study, an analysis of the genes co-expressed with *CEMIP* showed that the genes that were significantly positively related to *CEMIP* were mainly involved in biological processes such as the collagen catabolic process and collagen fibril organization. It was suggested that *CEMIP* may interact with these co-expressed genes and participate in the malignant biological behavior of GC cells by affecting the production of collagen.

*Helicobacter pylori* infection is thought to be the main cause of chronic gastritis, and the risk of GC in patients infected with *H. pylori* is about 1–3% [36,37]. The tumor microenvironment also plays an important role in cancer and inflammation. To the best of our knowledge, this was the first time that the relationship between *CEMIP* and immune cell infiltration was explored. The results showed that the expression of *CEMIP* was negatively correlated with the infiltration of B cells and CD4 + T cells. To further investigate the effect of *CEMIP* expression on the development of GC, we carried out several confirmatory *in vitro* experiments, and the results confirmed that the downregulation of *CEMIP* suppresses the proliferation and migration of GC cells and impairs the chemoresistance of GC cells to 5-FU.

Despite these results, our study still has some limitations. First, the limited number of gastric cancer samples in the cohorts we chose may lead to selection bias. Second, our method of identifying differentially expressed genes is mainly based on statistical analyses, therefore, other genes with biological significance may have been missed. To overcome the limitations of a retrospective design with a relatively small sample size, we suggest that a large-scale, multi-center, prospective study be performed to verify our results. Finally, more in-depth investigations regarding the mechanism of *CEMIP* function as well as animal studies are required lay a stronger theoretical foundation for the utility of this gene’s future clinical applications.

## Conclusion

In conclusion, WGCNA was used to identify DEGs in GC, and *CST1, CEMIP, COL8A1, PMEPA1*, and *MSLN* were identified as the hub genes. These genes can be used as novel prognostic markers or therapeutic targets in GC. It was observed that *CEMIP* plays a significant role in the development of GC and may potentially be used to guide the individualized treatment of patients with GC.

## Highlights

1. Six modules related to clinical characteristics of GC were determined by WGCNA.

2.Five hub genes, including CST1, CEMIP, COL8A1, PMEPA1 and MSLN were identified.

3.CEMIP down-regulation inhibits the biological behavior of cancer cells.
